# Supplementary material for: Shallow very-low-frequency earthquakes accompany slow slip events in the Nankai subduction zone
Source: Nat Commun. 2018 Mar 14;9:984. doi: 10.1038/s41467-018-03431-5 (PMC5852141; doi:10.1038/s41467-018-03431-5)
Supplement: Supplementary file 1 — Supplementary Information [file 41467_2018_3431_MOESM1_ESM.pdf]

Supplementary Information

Shallow very-low-frequency earthquakes accompany slow slip events in the Nankai subduction zone

Nakano et al.

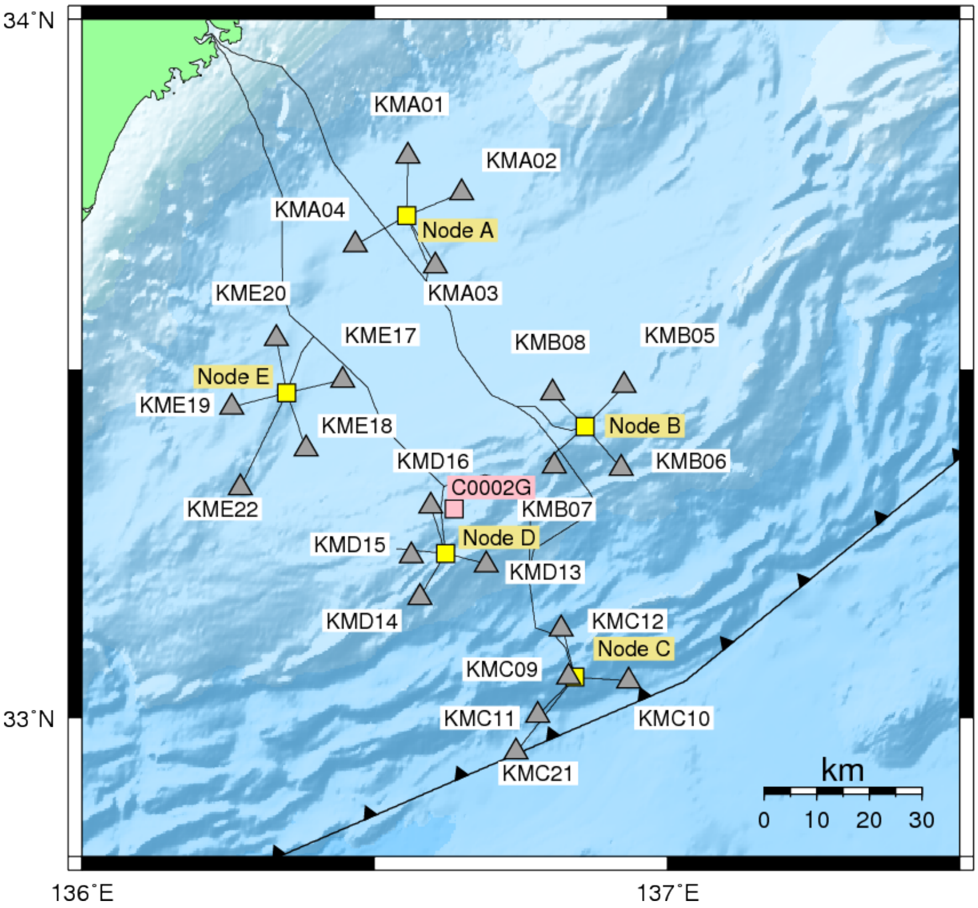

Supplementary Figure 1. DONET and borehole stations southeast of the Kii Peninsula. Gray triangles with a station code indicate DONET stations; black lines represent the optical fiber cables connecting the DONET stations and the land station; and the pink square is borehole station C0002G.

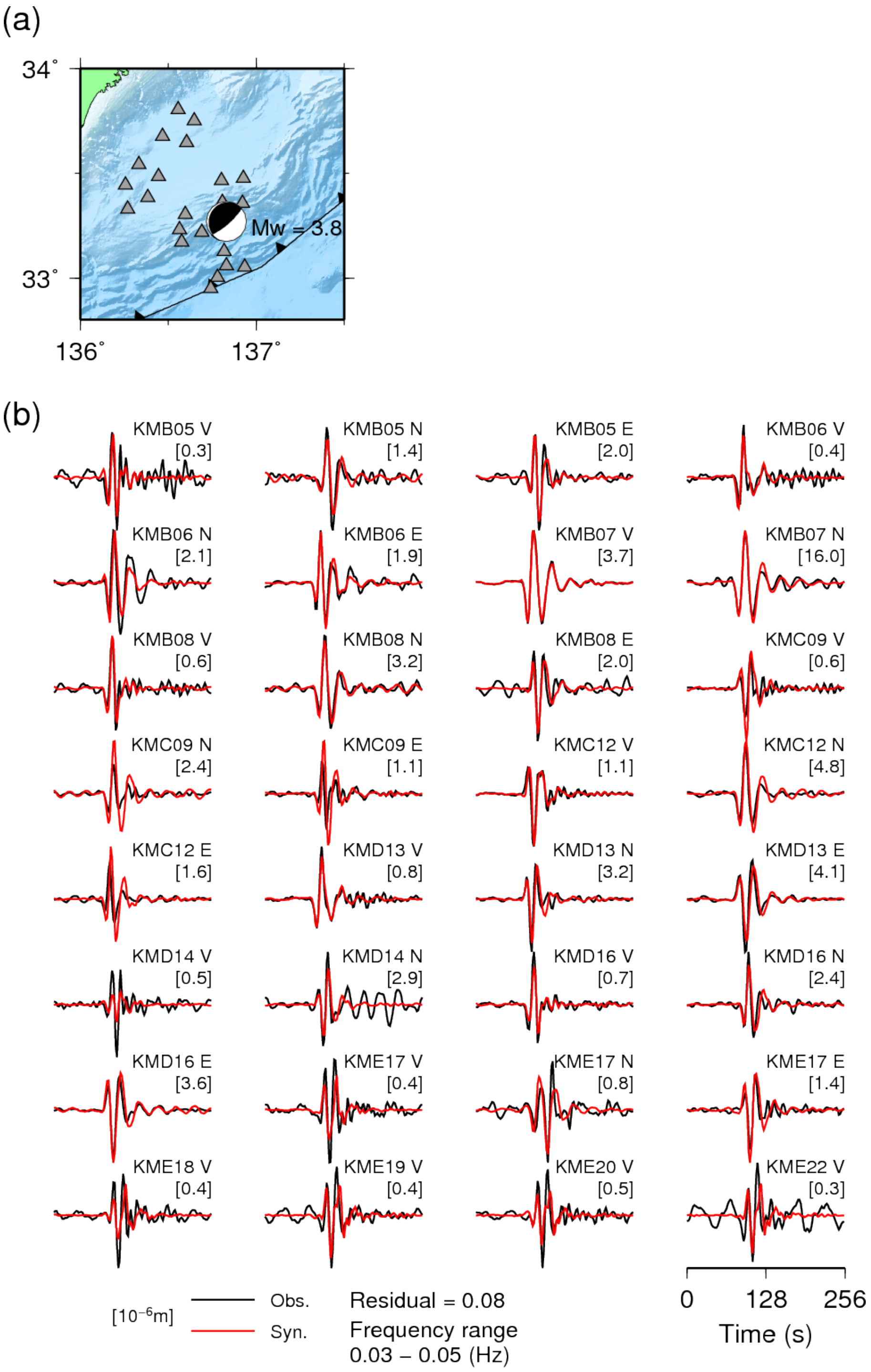

Supplementary Figure 2. An example of CMT analysis of a sVLFE. (a) Map showing the source location and focal mechanism plus locations of DONET stations (names appear in Supplementary Fig. 1). (b) Observed (black) and synthesized (red) displacement waveforms. The station code and component of motion are indicated above each waveform. Waveforms are normalized by the maximum value indicated in brackets.

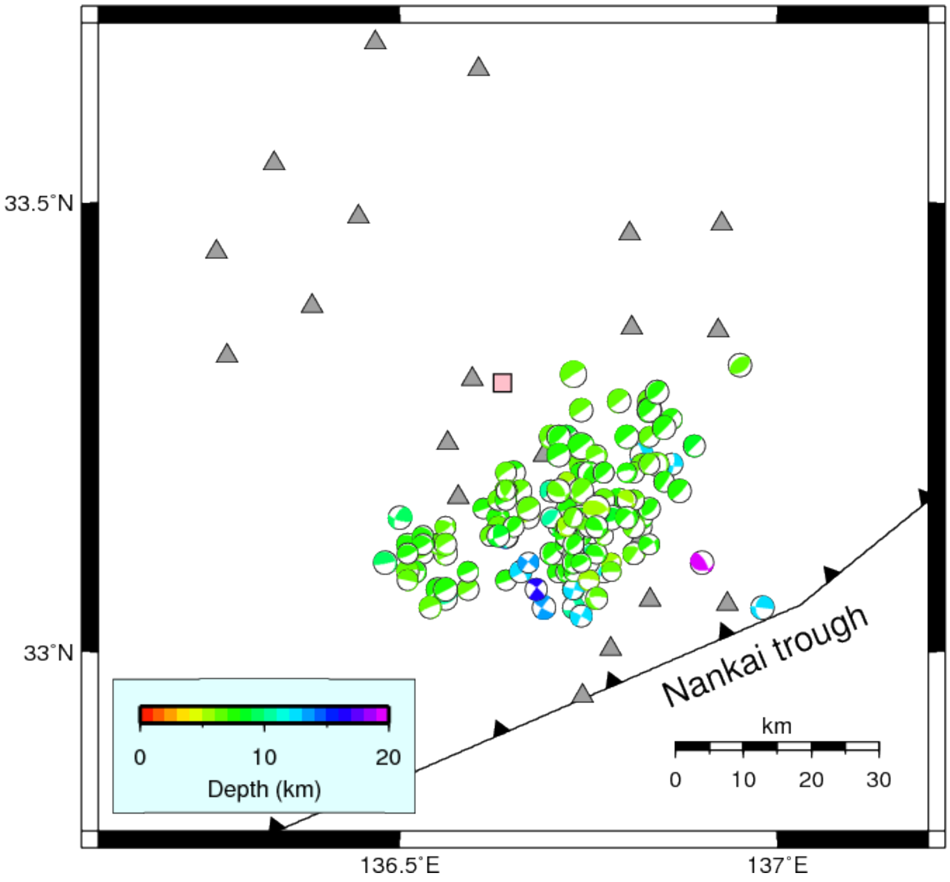

Supplementary Figure 3. Depth distribution of sVLFEs in 2016. Colors of sVLFE focal mechanism symbols represent source depth.

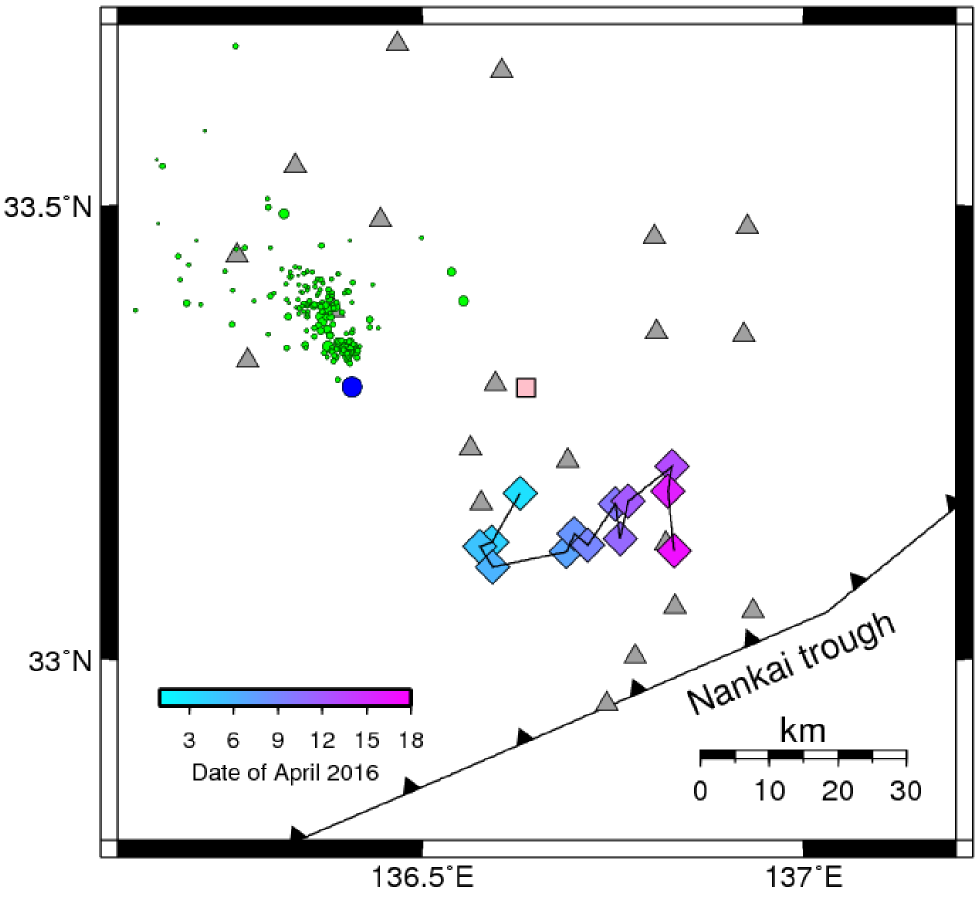

Supplementary Figure 4. Daily average locations of sVLFE sources (diamonds). Other symbols are the same as in Fig. 3.

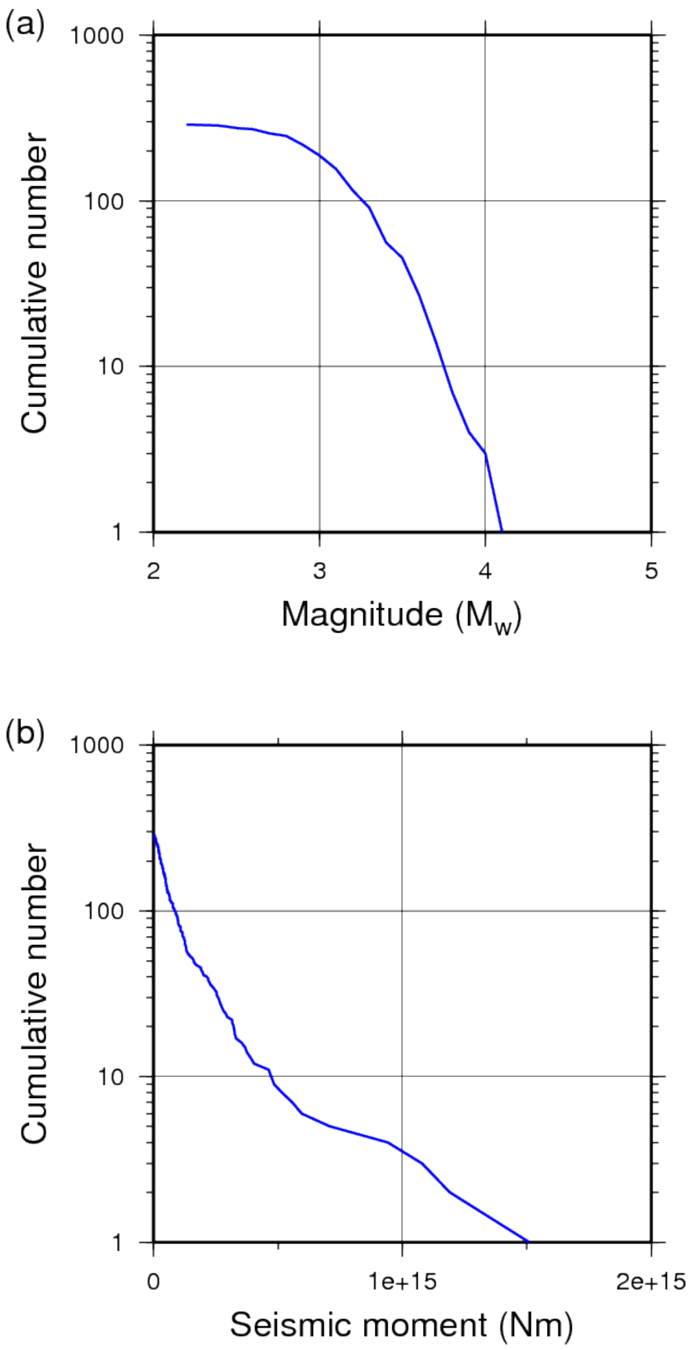

Supplementary Figure 5. Size distribution of sVLFEs in 2016. The cumulative number of events smaller than a given magnitude is plotted against (a) moment magnitude assuming a power-law (Gutenberg-Richter) distribution and (b) seismic moment assuming an exponential distribution.

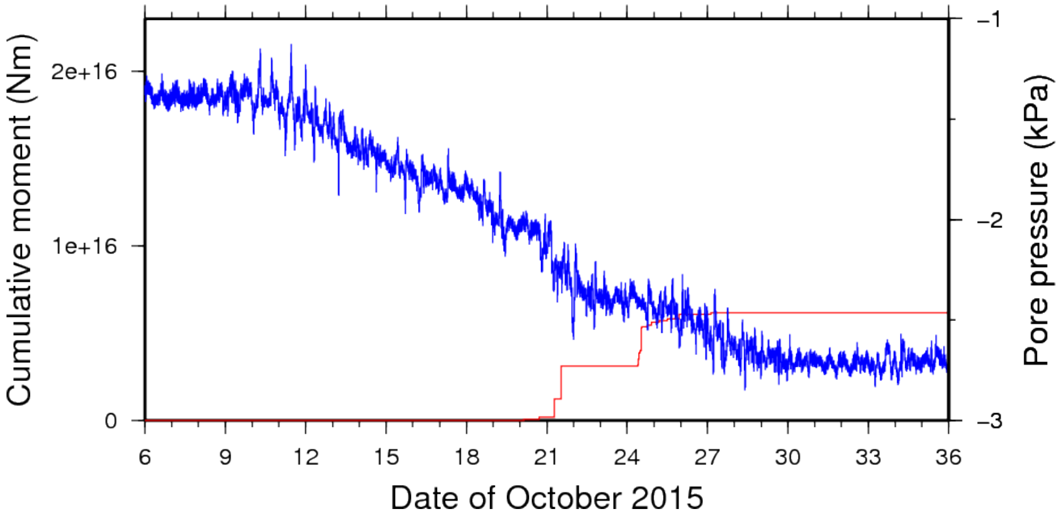

Supplementary Figure 6. Temporal evolution of sVLFE activity and borehole pore-pressure changes in the 2015 episode. Red curve shows the temporal evolution of sVLFE cumulative moment due to events larger than magnitude 3.0; blue curve shows borehole pore pressure.

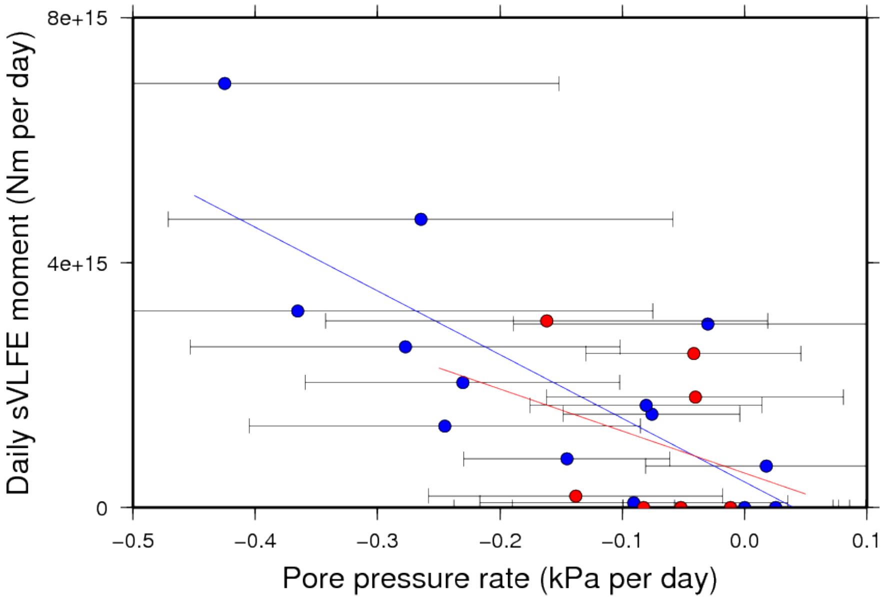

Supplementary Figure 7. Correlations of sVLFE moment release and pore-pressure changes. Daily moment releases due to sVLFEs are plotted against daily changes in borehole pore pressure. Red and blue circles represent sVLFEs in 2015 (20–30 October) and 2016, respectively, and lines with corresponding colors are linear fits to the data. Error bars of pore pressure rate are conventionally computed from the standard deviation of pore-pressure changes.

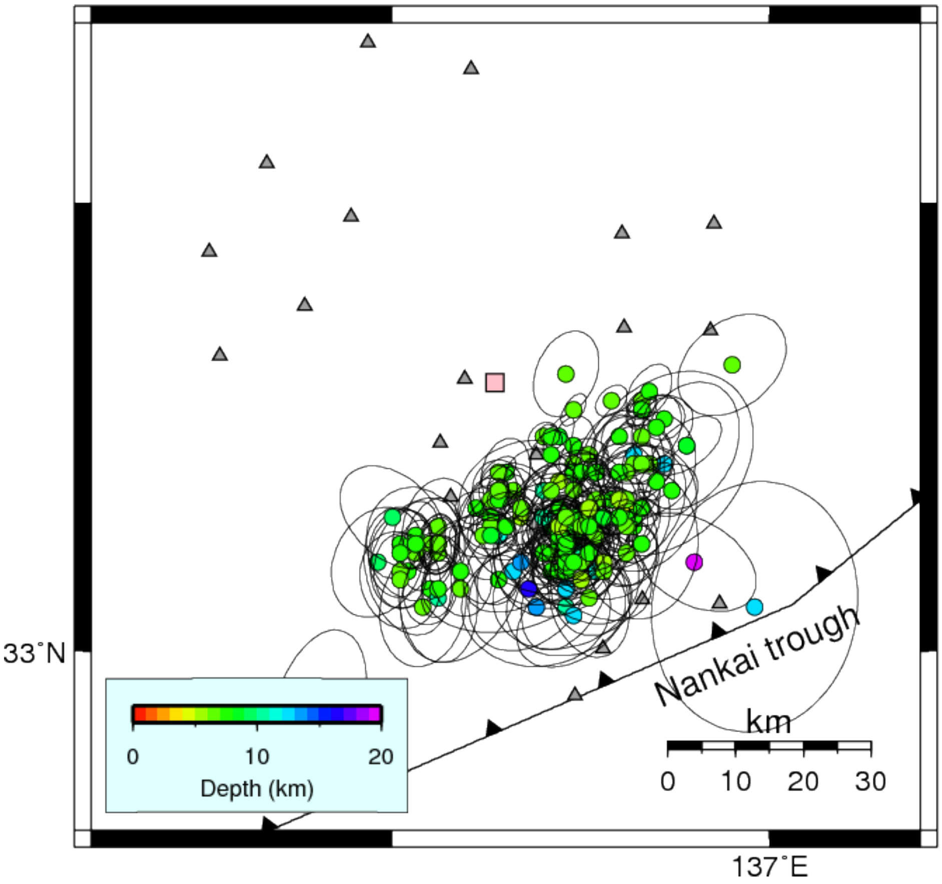

Supplementary Figure 8. Source location error of sVLFEs in 2016. Source location errors of events are shown by error ellipsoids centered at the best-fitting source locations.
